# The Paradox of Ingestion of Dietary Cholesterol in “Vegans”—Reply

**DOI:** 10.3390/nu9070786

**Published:** 2017-07-21

**Authors:** Peter Clarys, Tom Deliens, Inge Huybrechts, Peter Deriemaeker, Barbara Vanaelst, Willem De Keyzer, Marcel Hebbelinck, Patrick Mullie

**Affiliations:** 1 Faculty of Physical Education and Physiotherapy, Department of Human Biometrics and Biomechanics, Vrije Universiteit Brussel, Pleinlaan 2, 1050 Brussels, Belgium; tom.deliens@vub.be (T.D.); peter.deriemaeker@vub.be (P.D.); mhebbel@vub.be (M.H.); patrick.mullie@vub.be (P.M.); 2 Erasmus University College, Laerbeeklaan 121, 1090 Brussels, Belgium; 3 International Agency for Research on Cancer (IARC), Dietary Exposure Assessment Group, 150 Cours Albert Thomas, 69008 Lyon, France; Huybrechtsi@iarc.fr; 4 Department of Public Health, Ghent University, De Pintelaan 185, 9000 Ghent, Belgium; Barbara.Vanaelst@UGent.be (B.V.); willem.dekeyzer@hogent.be (W.D.K.); 5 Ghent University College, Vesalius, 9000 Ghent, Belgium; 6 International Prevention Research Institute (iPRI), 15 chemin du Saquin, Ecully, 69130 Lyon, France

In a comment on several articles on the vegan dietary pattern, Antoniazzi & Acosta-Navarro (2017) mentioned the paradox of the presence of dietary cholesterol as a nutritional component in the analysis of the vegan dietary pattern [1]. As dietary cholesterol is from animal origin, the latter nutritional component should not appear in the nutritional analysis of vegan dietary patterns. We agree with this statement and, in an answer to our colleagues, we mentioned the shortcomings of most actual food composition databases where often “a-likes” of vegan foods are used for the conversion of foods into macro- and micronutrients. The convinced vegan subject will surely control for the absence of animal components in all his/her foods [2]. However, food composition databases are not always updated and are often lacking adequate data for trends, new products, and less common products (such as vegan products).

In their comments, the authors propose to calculate each ingredient individually for each recipe to eliminate the presence of animal products. Although putting additional burden on an already time-consuming analysis, the latter may be a good solution, especially when additional information of the products can be captured to include in the diaries. For example, digital diaries collecting nutritional information by smart phone applications allow the inclusion of photographs, QR codes of products, and bar codes present on the package of food products. The latter can be used for recognition of the correct products to be analyzed. Moreover, due to the growing interest in healthy, ethical, and ecological food products, the number of available products is increasing, whilst some recent food composition databases used for analyses allow the addition of new products [3]. The latter option may be used to increase the number of vegan products in the used database, allowing researchers to choose a correct vegan product during the analysis procedure.

As also mentioned by Antoniazzi & Acosta-Navarro (2017) [1], clear definitions and criteria need to be used to describe the different forms of vegetarian diets [4]. In most of our studies, although classifications and criteria of the different types of diets are given, we still find subjects misclassified. For example, subjects declaring to be vegan may still be found to consume some animal products [5,6,7]. These subjects need to be excluded from the wrong classification and/or the researcher needs to switch the subjects after control to the correct classification. In particular, attention should be paid to the time people have adhered to a certain dietary pattern, since the pattern of a “new” or “searching” vegetarian may be different from a subject that has maintained vegetarian habits for several years. Moreover, (health) effects of the diet only become apparent after a certain time of adherence [8]. 

We thank Antoniazzi & Acosta-Navarro (2017) [1] for their comment and belief that taking into account their and the present suggestions will lead to research of higher quality on the different types of restricted diets.

Nevertheless, and awaiting better food composition databases, the existing databases can be useful for research linking dietary patterns to health outcomes. It must be clear that the presence of 10–40 mg of dietary cholesterol, as found in the more recent publications on vegan nutrition [9,10], will probably have an insignificant influence on any health outcome. This methodological issue may not be a barrier to implement further research. 
